# Fatal infection with emerging apicomplexan parasite *Hepatozoon silvestris* in a domestic cat

**DOI:** 10.1186/s13071-018-2992-4

**Published:** 2018-07-20

**Authors:** Kristel Kegler, Ursina Nufer, Amer Alic, Horst Posthaus, Philipp Olias, Walter Basso

**Affiliations:** 1 Institute of Animal Pathology, Vetsuisse Faculty, University of Bern, Bern, Switzerland; 2 Private Veterinary Clinic Dr. Usina Nufer, Meiringen, Switzerland; 3 Department of Pathology, Faculty of Veterinary Medicine, University of Sarajevo, Sarajevo, Bosnia and Herzegovina; 4 Institute of Parasitology, Vetsuisse Faculty, University of Bern, Bern, Switzerland

**Keywords:** Domestic cat, Myocarditis, *Hepatozoon silvestris*, Apicomplexa, Switzerland

## Abstract

**Background:**

*Hepatozoon silvestris* is an emerging apicomplexan parasite discovered in European wild cats from Bosnia and Herzegovina and blood samples of a domestic cat from Southern Italy in 2017. It has also been identified in *Ixodes ricinus* collected from a domestic cat in Wales, UK, in 2018. The clinical relevance, pathogenesis and epidemiology of this novel *Hepatozoon* species are not yet understood. Thus, the objective of this paper was to report and describe the first fatal case of an *H. silvestris* infection in a domestic cat.

**Results:**

The cat, which originated from Switzerland, died shortly after presenting clinical signs of lethargy, weakness and anorexia. At necropsy, no specific lesions were observed. Histopathology of the heart revealed a severe lympho-plasmacytic and histiocytic myocarditis. Mature and developing protozoal meronts morphologically compatible with *Hepatozoon* species were observed associated with the myocardial inflammation. No other lesions were present in any other organ evaluated, and the cat tested negative for retroviral and other immunosuppressive infectious agents. Polymerase chain reaction from the myocardium resulted in a specific amplicon of the *Hepatozoon 18S* rRNA gene. Sequencing and BLAST analysis revealed 100% sequence identity with *H. silvestris.*

**Conclusions:**

The severity of the infection with fatal outcome in an otherwise healthy animal suggests a high virulence of *H. silvestris* for domestic cats. The presence of this emerging parasite in a domestic cat in Switzerland with no travel history provides further evidence for a geographical distribution throughout Europe.

## Background

*Hepatozoon* species are apicomplexan parasites (family Hepatozoidae) with more than 340 species recognized [1, 2]. Unlike most vector-borne protozoan pathogens which are transmitted during a blood meal, *Hepatozoon* infections take place when the intermediate vertebrate host ingests the definitive host, an invertebrate, containing mature oocysts [3]. Other modes of transmission, such as transplacental or by predation of infected intermediate hosts, have been reported [4–6]. Recent studies involving molecular diagnostic techniques identified two distinct species, *H. americanum* and *H. canis*, infecting wild and domestic carnivores [7–11], while *H. felis* was recognized in wild and domestic felids [6, 10, 12, 13]. Studies on domestic cats from European countries including Italy [14], France [15], Spain [16] and Portugal [17] identified *H. felis* in blood samples. In 2017, *Hepatozoon silvestris* was described as novel species in European wild cats (*Felis silvestris silvestris*) from Bosnia and Herzegovina [13]. Shortly thereafter, *H. silvestris* DNA was amplified and sequenced from blood samples of a domestic cat from southern Italy [14] and *Ixodes ricinus* ticks collected from a domestic cat in Wales, UK [18].

The pathogenic potential of feline hepatozoonosis in general is poorly understood. *Hepatozoon felis* infection in domestic cats is considered subclinical with no apparent inflammatory response associated with the presence of meronts in muscle tissue [2, 6, 8, 16, 19]. Similar to *H. felis*, *H. silvestris* infects myocardial and skeletal muscles in European wild cats and has been found to be associated with only a minimal inflammatory response [13]. Here we report the first fatal case of *H. silvestris* infection associated with a severe myocarditis in a domestic cat.

## Methods

At necropsy, representative samples of the heart, lung, liver, kidneys, spleen, lymph nodes, bone marrow, stomach, intestine and brain were collected, fixed in 10% neutral buffered formalin, processed routinely and embedded in paraffin wax. Sections of 3 μm were stained with haematoxylin and eosin (HE). Polymerase chain reaction (PCR) to identify *Hepatozoon* species was performed using the primers H14Hepa18SFw and H14Hepa18SRv [20], which amplify a fragment of the *18S* rRNA gene of *Hepatozoon* spp. For this, DNA was extracted from 20 μm sections of formalin-fixed and paraffin-embedded (FFPE) tissue of the heart as previously described [21]. As a positive control, DNA extracted from FFPE heart sections of a naturally infected European wild cat [13] was used. DNA amplification was conducted under the following conditions: 95 °C for 15 min, followed by 35 cycles at 58 °C for 1 min, 72 °C for 1 min and 95 °C for 1 min, and a final extension at 72 °C for 5 min. The amplified PCR product was purified using a commercial kit (DNA Clean & Concentrator-5 Zymo Research, Irvine, USA) and subsequently sequenced (Microsynth, Balgach, Switzerland) in both directions with the same primers as used in the PCR. An additional real time PCR targeting the *Toxoplasma gondii B1* gene was carried out as previously described [22]. Alignment of the sequence of *Hepatozoon* species was performed using CLUSTALW incorporated in the MEGA7 software package [23]. Unrooted phylogenetic networks were generated with SplitsTree4 (v. 4.14.6 [24]) using a neighbor-net method. jModelTest2 [25] identified HKY+I as optimal substitution model.

## Results

A 5-year-old neutered male European shorthair cat from the municipality of Hofstetten bei Brienz (approximate location: 46°45'17"N 8°4'38"E) was presented to a private veterinary clinic in Meiringen, Switzerland in September 2017 with clinical signs of lethargy, weakness and anorexia. At clinical examination no ectoparasites were observed. The cat had free outdoor access, never traveled to another country, and was regularly dewormed, and vaccinated against feline panleukopenia, feline viral rhinotracheitis and feline calicivirus (FCV). Blood samples were collected and sent to a private laboratory for hematologic and biochemical analyses, and feline immunodeficiency virus (FIV) and feline leukemia virus (FeLV) testing. The cat received intravenous infusions but died shortly after admission and was sent to the Institute of Veterinary Pathology of the Vetsuisse Faculty in Bern, Switzerland for complete post mortem evaluation. Hematologic and biochemical analysis of blood samples revealed mild thrombocytopenia (159 K/μl; reference range 175–600 K/μl) and slightly increased pancreatic lipase enzyme (1446 U/l; reference range 100–1400 U/l). Red blood cell (RBC) and white blood cell (WBC) count, as well as standard biochemical parameters were within normal range. Additionally, blood samples tested negative for FIV and FeLV. At necropsy, the animal was in a good nutritional state. Gross lesions were unspecific and consisted of a mild generalized lymphadenomegaly, presence of approximately 10 ml of serous transudate within the thoracic cavity, mild pulmonary edema, and mild diffuse myocardial pallor of both ventricles of the heart.

**Fig. 1:**
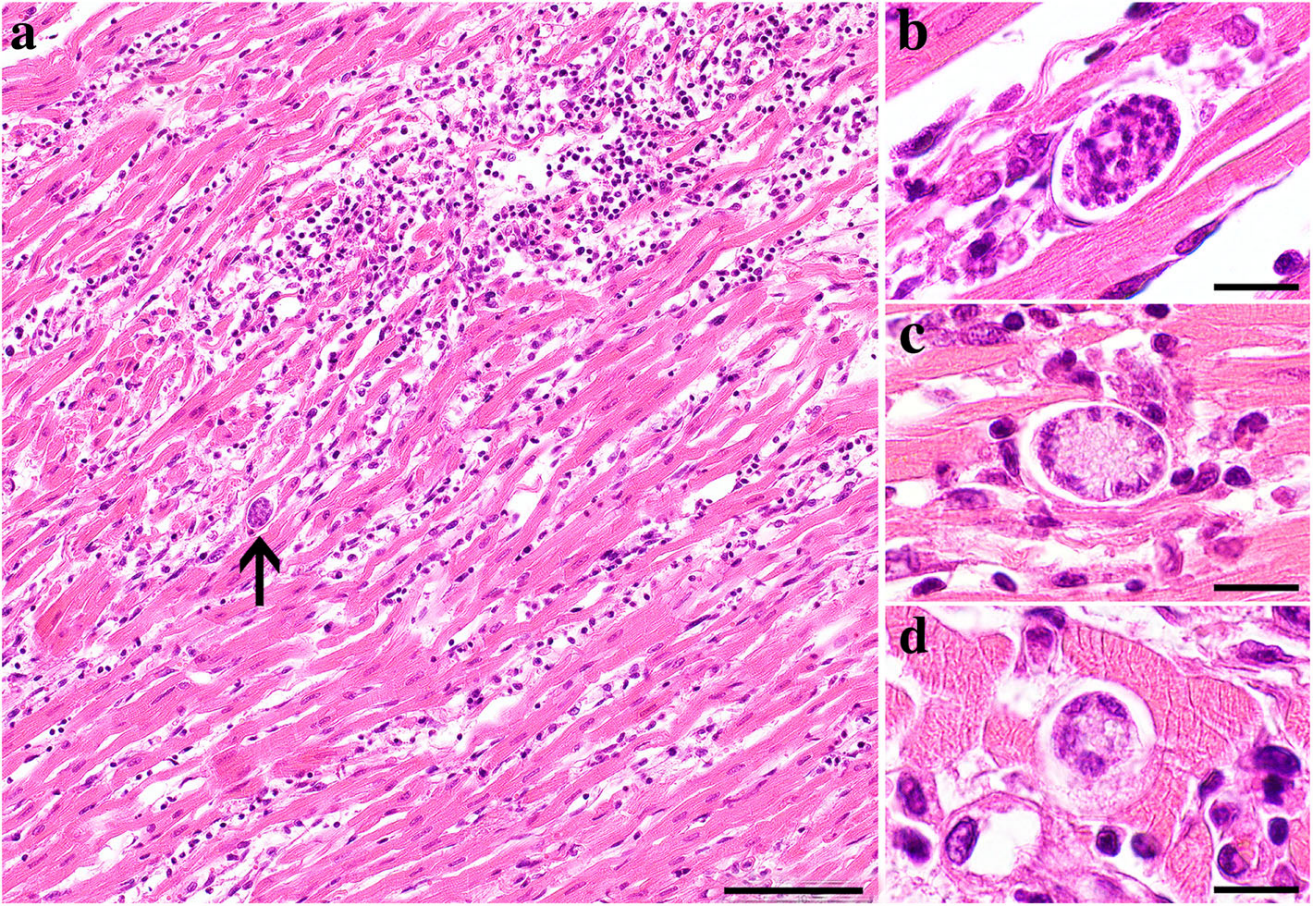
Photomicrographs of the myocardium of the domestic cat infected with *H. silvestris*. **a** HE stained section of the myocardium severely infiltrated by lymphocytes, plasma cells and macrophages, with associated myofiber degeneration and necrosis. Note the presence of an intralesional mature meront (arrow). **b** Higher magnification of a mature meront filled with numerous merozoites. **c** Developing wheel-spoke shaped meront with micromerozoites arranged in a circle along the wall. **d** Developing meront with elongated circularly aligned macromerozoites. *Scale-bars*: **a**, 100 μm; **b**-**d**, 20 μm

Histopathology of the heart disclosed a severe multifocal to coalescing lympho-plasmacytic and histocytic myocarditis associated with cardiomyocyte degeneration and necrosis, few neutrophils, moderate interstitial edema and multifocal hemorrhages (Fig. 1a). Myocardial lesions were disseminated throughout both ventricles, the interventricular septum and the atria. The epicardium and endocardium were moderately and diffusely expanded by the same inflammatory infiltrate and marked edema. Protozoal meronts measuring up to 32 × 22 μm and enveloped by an up to 1 μm thick capsule were associated with the inflammatory lesions (Fig. 1a, arrow). Mature and two different types of developing meronts were observed. Mature meronts (Fig. 1b) were characterized by numerous, round, 2–4 μm in diameter irregularly scattered merozoites. The first type of developing meront contained approximately 20–30 small, up to 4 × 2 μm, oval micromerozoites which were circularly arranged alongside the meront wall (Fig. 1c). The second type contained 2–8 larger and elongated macromerozoites, up to 6 × 3 μm, which were dispersed within the meront or circularly arranged alongside the meront wall (Fig. 1d). Additional findings included moderate pulmonary alveolar histiocytosis and interstitial edema, acute centrilobular hepatocellular degeneration and mild hepatic extramedullary hematopoiesis. Mild follicular hyperplasia was noted in tracheobronchial and mesenteric lymph nodes. No histopathological lesions were observed in the spleen, pancreas, kidneys, stomach, small and large intestines, brain and bone marrow.

**Fig. 2:**
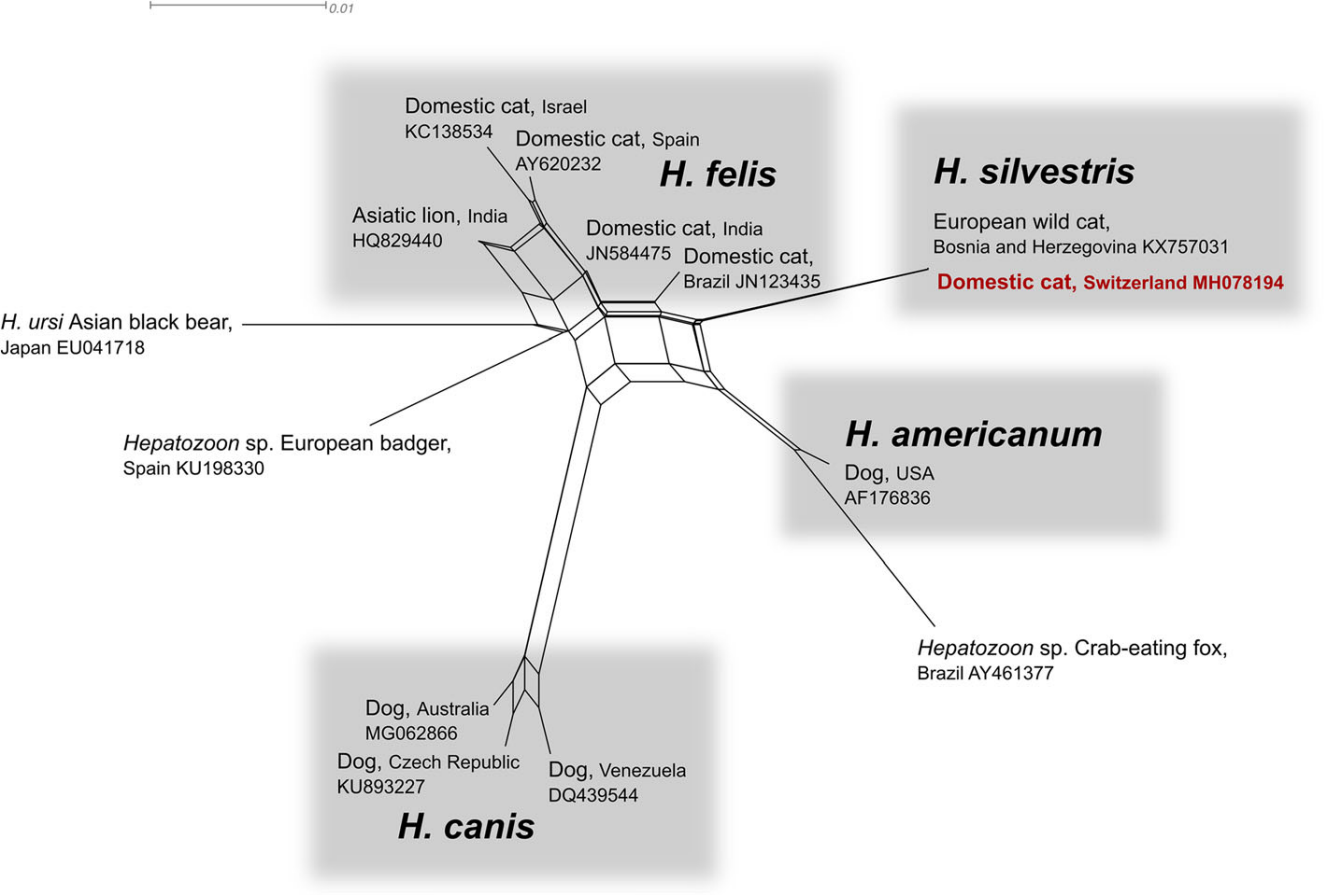
Phylogenetic network of a trimmed 546 bp fragment of the *18S* rRNA gene of *Hepatozoon* spp. as calculated by the neighbor-net algorithm in SplitsTree4. *Hepatozoon silvestris* and other species infecting domestic carnivores are shaded in grey. Note that the *Hepatozoon 18S* sequence fragment derived from the infected myocardium of the domestic cat described here is identical to the sequence from a European wild cat from Bosnia-Herzegovina

Based on the presence of intralesional meronts in the heart muscle, the preliminary diagnosis of hepatozoonosis was made. *Hepatozoon* spp. DNA was amplified from the heart with genus specific primers. A PCR for *T. gondii* gave negative results. BLAST analysis and comparison of amplified 572 bp (GenBank: MH078194) with publically available sequences disclosed 100% sequence identity with *H. silvestris* from European wild cats (KX757032) and a feline blood sample from Italy (KY649445). The sequence similarity to the closest *H. felis* genotype (JN123435) was 96%. The results of the phylogenetic analysis showed a close but distant relationship of *H. silvestris* to multiple *H. felis* genotypes incorporated in the network calculation (Fig. 2).

## Discussion

To our knowledge, this is the first report of a fatal myocarditis associated with the emerging parasite *H. silvestris* in a domestic cat. So far, epidemiological data on *H. silvestris* were limited to European wild cats from Bosnia and Herzegovina [13, 20] and a report of a positive feline blood sample from southern Italy [14]. *Hepatozoon felis* was considered the most common species infecting wild and domestic felids worldwide [6, 14, 16, 26–28]. In Europe, infections of cats with *H. felis* mainly occur in Mediterranean countries where hematophagous arthropods are more abundant [8, 12, 14, 16]. The autochthonous case of an *H. silvestris* infection in Switzerland described here expands the geographical range of this emerging parasite.

The life-cycle and mode of transmission of feline hepatozoonosis is unknown. Recent studies were unable to establish a correlation between the infestation with arthropods and *H. felis* infection [8, 16]. It is also noteworthy that haemolymph smears of ticks collected from *H. silvestris* infected European wild cats failed to demonstrate the presence of *Hepatozoon* oocysts [13]. In contrast, *I. ricinus* collected from a domestic cat in Wales, UK, in 2018 tested positive for *H. silvestris* PCR analysis [18]. It is therefore tempting to speculate that *I. ricinus* plays a role in the transmission of *H. silvestris*, potentially in addition to a transmission route by the ingestion of infected prey [5, 13]. Epidemiological studies are needed to identify further invertebrate or vertebrate hosts involved in the life-cycle of this parasite.

The inflammatory lesions associated with *H. silvestris* reported here caused fatal heart failure in the domestic cat. The pulmonary and hepatic lesions most likely resulted from an acute cardiac insufficiency due to the myocardial lesions. Interestingly, the inflammatory response associated with *H. silvestris* meronts in the myocardium of European wild cats was very mild [13]. The closest relative, *H. felis*, is considered of low virulence and associated mostly with subclinical or mild clinical signs such as lethargy, fever, weakness and lymphadenopathy, and inconsistent blood and biochemical abnormalities [6, 8, 16, 19]. Elevated levels of muscular activity enzymes including serum lactate dehydrogenase (LDH) and creatine phosphokinase (CK) are described in a small number of cats infected with *H. felis* [8]. In the present case, the activity of muscular enzymes was not evaluated. Since PCR is widely used to detect *H. felis* in blood samples of apparently healthy domestic cats, further studies are needed to assess this less invasive method, together with a muscular enzymatic activity profile, as diagnostic tool in clinical cases of *H. silvestris* infection.

Immunosuppressive co-infections have been proposed to contribute to the severity of feline hepatozoonosis [8, 19, 29]. The cat presented here tested negative for feline immunocompromising retroviruses (FeLV and FIV) of cats and no lesions suggestive of any other concurrent immunosuppressive disease or infection were diagnosed. Our findings therefore strongly suggest that *H. silvestris* can cause fatal infections in otherwise healthy domestic cats.

## Conclusions

The case of a *H. silvestris* infection in a domestic cat reported here demonstrates the presence of this emerging apicomplexan parasite in Switzerland. More importantly, the parasite-associated fatal myocarditis suggests a high virulence of *H. silvestris* for domestic cats [13]. Further epidemiological and experimental studies are needed to identify transmission routes, reservoir, definitive hosts and to elucidate the pathogenicity of this newly identified pathogen.

## Abbreviations

DNA: deoxyribonucleic acid
FFPE: formalin-fixed and paraffin-embedded tissue
PCR: polymerase chain reaction
FCV: feline calicivirus
FIV: feline immunodeficiency virus
FeLV: feline leukemia virus
RBC: red blood cell
WBC: white blood cell
LDH: lactate dehydrogenase
CK: creatine phosphokinase
